# *Rosmarinus officinalis* L. Essential Oils Impact on the Microbiological and Oxidative Stability of Sarshir (Kaymak)

**DOI:** 10.3390/molecules28104206

**Published:** 2023-05-20

**Authors:** Seyed Mohammad Bagher Hashemi, Aliakbar Gholamhosseinpour, Francisco J. Barba

**Affiliations:** 1 Department of Food Science and Technology, Faculty of Agriculture, Fasa University, Fasa 74616-86131, Iran; 2 Department of Food Science and Technology, Faculty of Agriculture, Jahrom University, Jahrom 74131-88941, Iran; 3 Department of Preventive Medicine and Public Health, Food Sciences, Toxicology and Forensic Medicine, Faculty of Pharmacy, Universitat de València, Avda. Vicent Andrés Estellés, s/n, 46100 Burjassot, València, Spain

**Keywords:** antibacterial activity, antioxidant capacity, essential oil, *Rosmarinus officinalis* L.

## Abstract

This study investigated the effect of *Rosmarinus officinalis* L. essential oil, REO (one, two and three percent) on the microbiological and oxidative stability of Sarshir during 20 days of refrigerated storage (4 °C). Initially, the chemical composition (gas chromatography/mass spectrometry, GC/MS), antimicrobial (paper disc diffusion) and antioxidant (DPPH) properties of REO were evaluated. Then, the microbial safety, oxidative stability (peroxide and anisidine values) and overall acceptability of the product after addition of REO to Sarshir and the subsequent storage period were determined. According to GC/MS analysis, the major components of REO were α-pinene (24.6%), 1,8-cineole (14.1%), camphor (13.5%), camphene (8.1%) and limonene (6.1%), respectively. Moreover, it was also found that *Limosilactobacillus fermentum* (inhibition zone (IZ) of 23.5 mm) and *Salmonella* Typhi (IZ of 16.4 mm) were the most sensitive and resistant spoilage and pathogenic bacteria against REO, respectively. In addition, the half-maximal inhibitory concentration (IC50) of the REO was measured at 24.8 mg/mL, while the IC50 value of butylated hydroxytoluene (BHT) was 16.6 mg/mL. The highest and lowest bacterial populations were detected in the control and the sample containing 3% REO, respectively. The control had the highest extent of lipid oxidation, while the lowest peroxide and anisidine values were measured in Sarshir containing 3% REO.

## 1. Introduction

The undesirable growth of microorganisms, together with the occurrence of oxidation processes (especially lipid oxidation), cause spoilage, off-flavours, inappropriate changes and ultimately the waste of food products. The problems related to the negative effects of some synthetic antimicrobial and antioxidant compounds on the health of consumers have led to an increase in interest in the use of natural compounds that can increase the shelf life of food without harmful effects [1]. Therefore, the use of natural antimicrobials and antioxidants constitutes a useful, safe and effective approach to reduce these risks [2].

Over the last decades, an increased growth in the use of traditional herbs and medicinal plants has been observed due to their high content of natural compounds with biological properties [3]. Essential oils (EOs) are natural aromatic compounds obtained from various plants. The extended biological properties of these compounds have led to their use as flavouring, antioxidant, antimicrobial and antiallergenic agents [4,5]. It has also been reported that essential oils have the potential to treat COVID-19 disease. These agents can prevent SARS-CoV-2 infection by targeting its receptors on cells [6].

Rosemary (*Rosmarinus officinalis* L.) is an aromatic medicinal plant belonging to the Lamiaceae family and grows wild in most of the Mediterranean countries. In addition to its clinical applications, rosemary is used in the food industry to enhance flavour, inhibit oxidation, and reduce microbial contamination. REO is an almost colourless to pale yellow liquid with a distinct and pleasant smell [7,8]. The antioxidant and antimicrobial activities of rosemary are mainly attributed to its phenolic compounds, including rosmarinic acid, carnosoic acid, rosmanol, carnosol, and epirosmanol [8,9]. In general, it has been reported that the antioxidant activities have a high correlation with the content of flavonoids and phenols [10].

EOs can have a negative effect on the organoleptic properties of products containing them, due to their volatility and strong flavour, which should be considered in the final formulation of the product. Achieving the optimal dose of EO and using the microencapsulation technique are among the strategies used to overcome this limitation [4]. Dairy and meat products are interesting and promising food targets to evaluate and exploit the antimicrobial characteristics of EOs due to the fewer expected negative effects on their sensory properties [11]. Sarshir (Kaymak) is a type of dairy product that is widely consumed in Iran, Turkey and Eastern European countries. To produce this product, first, the milk is gently boiled and then gradually cooled to form a layer on the surface of the milk. Finally, the layer is separated. Sarshir is sensitive to microbial contamination and is very susceptible to oxidation due to its high fat content [12]. The maximum shelf life of Sarshir, especially the traditional varieties, is 4 to 7 days while maintaining the appropriate quality characteristics [13].

Considering that Sarshir is mainly produced traditionally, microbial contamination of the product seems reasonable. Various studies have reported the contamination of Sarshir with aerobic mesophilic bacteria, coliform, salmonella, shigella, staphylococcus, yeast and mould. *E. coli* was also detected in some samples. Sarshir’s microbial contamination can be improved using modern techniques and equipment, and implementing good manufacturing practices (GMP), including HACCP [13,14]. Moreover, the packaging materials should be hygienically suitable, and the final product should be kept under the cold chain until it is released to the market [13].

Lactic fermentation and the use of essential oils are among the strategies that have recently been considered to increase microbial safety and improve the quality of Sarshir. In a study, Hashemi et al. [12] used three probiotic strains of *L. plantarum* (LP3, AF1, and LU5) to ferment Sarshir and their effects on the antibacterial and antioxidant activities of Sarshir were examined. Based on the results, LAB strains had an inhibitory effect on the pathogens *P. aeruginosa*, *E. coli* O157: H7, *B. cereus* and *S. aureus*. In addition, the fermentation of Sarshir by *L. plantarum* strains, especially LP3, resulted in beneficial changes in radical scavenging activity, peroxide, anisidine, and carbonyl values, indicating that these strains can help improve the quality and oxidative stability of Sarshir. In another study, Albay and Şimşek [15] used cinnamon powder (0.3%) and cinnamon essential oil (0.002% and 0.005%) in the production of Sarshir. They stated that these ingredients masked the bad milk odour and sour odour and taste defects from milk. According to the reported results, the control sample, cream containing cinnamon powder and cream with 1% cinnamon essential oil had the highest sensory acceptance. It was also concluded that adding 0.3% of cinnamon powder and 0.005% of cinnamon essential oil can improve the functional properties of the product, increase the sensory acceptability and provide a different variety of the product.

Considering the antibacterial and antioxidant capacities of *Rosmarinus officinalis* L. EO (REO) and the fact that Sarshir is a perishable dairy product, in this research, the potential of using REO to promote the microbial safety and oxidative stability of Sarshir was investigated.

## 2. Results and Discussion

In the first step, we extracted REO and after determining the chemical composition, we measured the antimicrobial and antioxidant properties of the pure EO. In the second step, REO was added to the Sarshir in given concentrations to determine what effects it would have on the microbial characteristics and oxidative stability of the product during the storage period.

### 2.1. Chemical Composition of REO

After performing the GC/MS analyses, fifty-one compounds were detected in REO, comprising ~97% of the oil (Table 1). The order of essential oil compounds is based on their elution from the capillary column. The major constituents of the REO were α-pinene (24.6%), 1,8-cineole (14.1%), camphor (13.5%), camphene (8.1%) and limonene (6.1%), respectively. Compared to previous studies, Bajalan et al. [16] reported that the most important compounds of REO were α-pinene (14.2–21.4%), 1,8-cineole (3.3–28.3%) and camphor (1.6–25.3%). On the other hand, the predominant constituents of REO in the investigation of Oualdi et al. [17] were: 1,8-cineole (42.3–53.6%), α-pinene (11.6–12.3%) and camphor (9.6–10.5%). Variation in the chemical composition of EOs in different research has been attributed to differences in cultivars, geographical origin, harvesting season, environmental conditions, sampling and extraction methods [18,19].

### 2.2. Antibacterial Activity of REO

**Figure 1:**
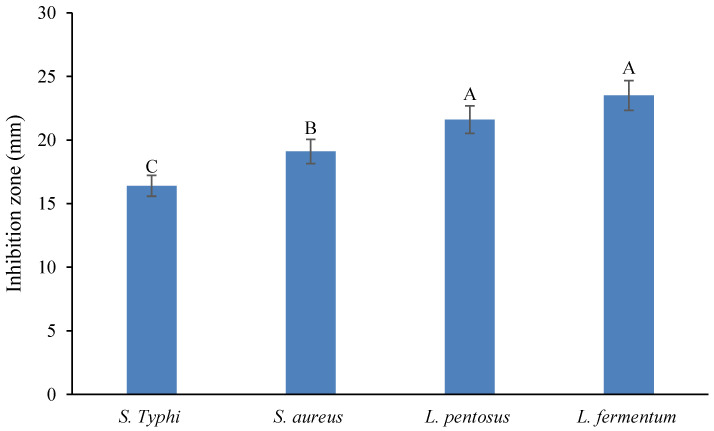
The antibacterial activity of REO. Columns with various capital letters are significantly different (*p* < 0.05).

The antibacterial activities of REO are shown in Figure 1. According to the figure, *L. fermentum* (IZ of 23.5 mm) and *S.* Typhi (IZ of 16.4 mm) had the highest and lowest sensitivity to REO, respectively. In general, gram-positive bacteria (*L. fermentum*, *Lactiplantibacillus pentosus* and *S. aureus*) exhibited larger inhibition zones than *S.* Typhi as a gram-negative bacterium.

Saleh et al.’s observations also indicated a stronger inhibitory effect of REO on *Staphylococcus aureus* with an IZ of 30 mm compared to *E. coli* (IZ of 25 mm) [20]. Additionally, a significant difference (*p* ≤ 0.05) was found between the inhibitory effect of REO on the pathogens (*S.* Typhi and *S. aureus*), while this difference was not significant for spoilage bacteria (*L. fermentum* and *L. pentosus*).

Phenolic compounds and small terpenoids present in EOs play an important role in their antibacterial activity. The dominant phenolic compounds of REO, including alpha-pinene, 1,8-cineole, carvacrol, camphor, borneol and bata-caryophyllene, are the main ones responsible for its antibacterial activity [20,21].

Due to their hydrophobic properties, EOs bind to the bacterial cell membrane lipids, increasing cell permeability. The release of ions and other cellular components caused by the permeability of the cell membrane eventually causes the death of the microorganism [22]. The presence of an outer membrane other than the thin layer of peptidoglycan makes gram-negative bacteria have a more complex cell wall compared to gram-positive types [23]. Considering the protective role of this outer membrane, the lower sensitivity of these bacteria to the antibacterial effects of EOs compared to gram-positive bacteria is expected [24].

### 2.3. Antioxidant Activity of REO

**Figure 2:**
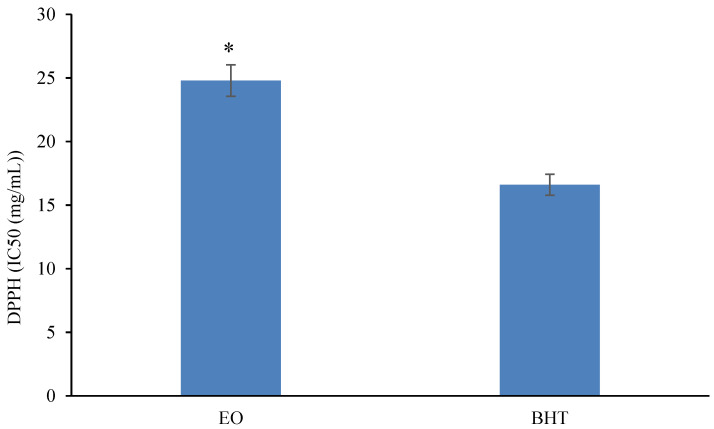
The antioxidant activity of REO compared to BHT (* Statistically significant at *p* ≤ 0.05).

The EC50 parameter is widely applied to determine the free radical scavenging activity. The lower the EC50 value, the higher the antioxidant capacity [25]. Figure 2 shows the antioxidant activity (based on the DPPH scavenging assay) of REO compared to BHT as a synthetic antioxidant. Accordingly, the free radical scavenging activity of the REO was measured at 24.8 mg/mL, while the IC50 value of BHT was 16.6 mg/mL. In other words, the BHT antioxidant capacity was significantly (*p* ≤ 0.05) higher than that of the REO. Our results were concordant with the results of Hashemi et al. [26] and Goudoum, Abdou, Ngamo, Ngassoum and Mbofung [25], who found that BHT had a higher free radical scavenging activity than the studied EOs. The terpenoid compounds of the REO are the main reason for its high antioxidant capacity. Among the most effective antioxidant compounds of rosemary, cyclic diterpene diphenols, carnosol and carnosolic acid have been mentioned. Moreover, a compound like 1,8-cineole, at 14.1% in this essential oil, can contribute to the high antioxidant activity of REO [5,8].

### 2.4. Antibacterial Activity of REO in Sarshir

**Figure 3:**
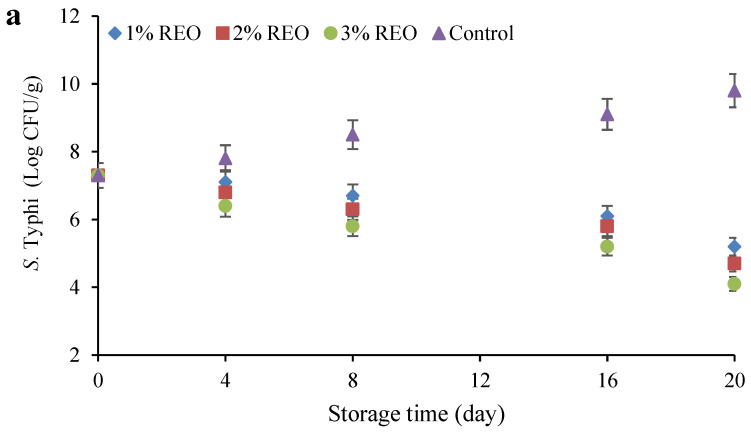

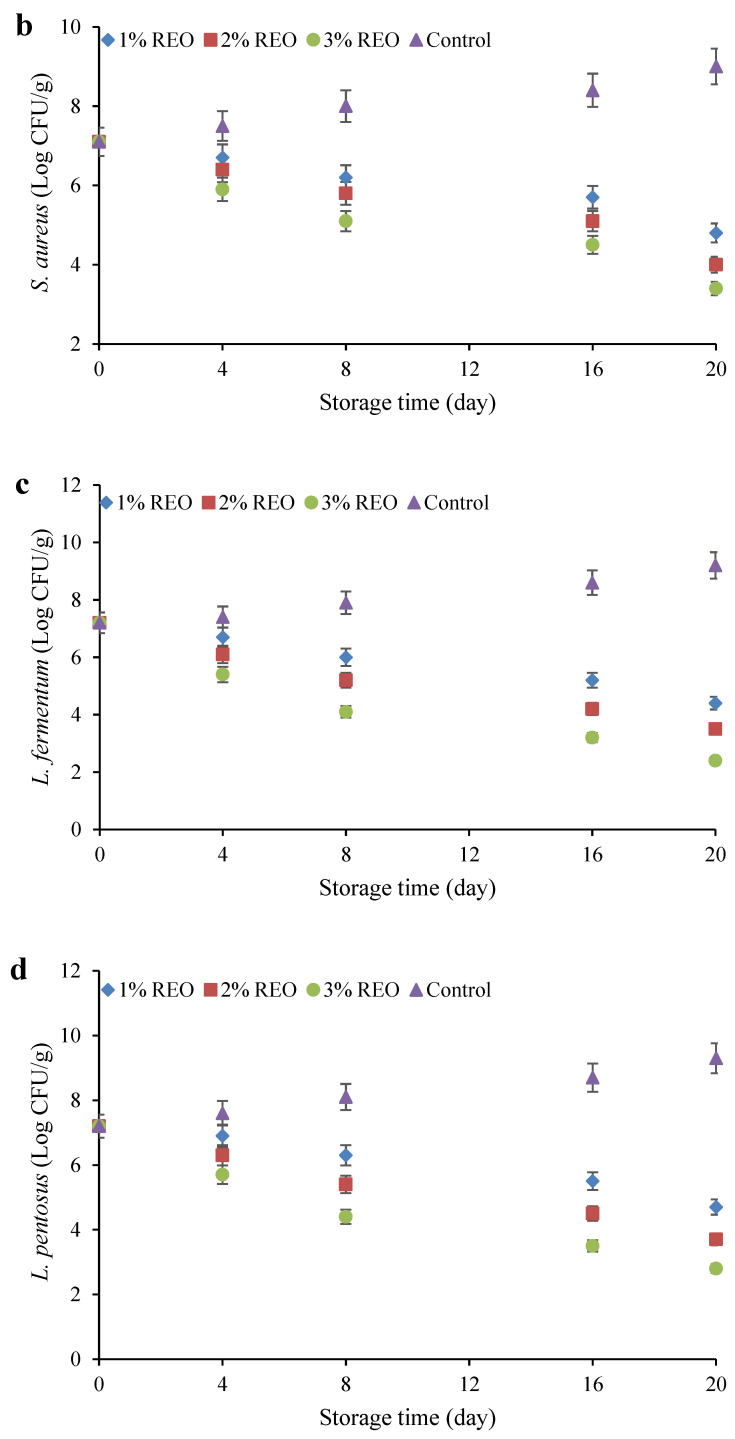
The antimicrobial effects of different concentrations of REO on pathogenic (*S.* Typhi (**a**) and *S. aureus* (**b**)) and spoilage bacteria (*L. fermentum* (**c**) and *L. pentosus* (**d**)) in Sarshir during the cold storage period.

The antimicrobial effects of different REO concentrations on food-borne pathogens (*S.* Typhi and *S. aureus*) and spoilage bacteria (*L. fermentum* and *L. pentosus*) during the cold storage period are shown in Figure 3. The number of *S.* Typhi in the control increased significantly (*p* ≤ 0.05) from 7.3 (Log CFU/g) on day zero to 9.8 (Log CFU/g) on day 20, while in samples containing REO, the count of this pathogen decreased with increasing storage time (Figure 3a). The control had the highest number and the sample containing 3% REO had the lowest number of *S.* Typhi. As shown in Figure 3b, the count of *S. aureus* in the control also increased significantly (*p* ≤ 0.05) during refrigeration (ranging from 7.1 to 9 (Log CFU/g)), while its numbers in the samples containing different concentrations of REO had a downward trend. The number of *S. aureus* in the control and the sample containing 3% REO was considerably (*p* ≤ 0.05) higher and lower than its number in other samples, respectively. With increasing storage time, the population of *L. fermentum* in Sarshir without REO increased significantly (*p* ≤ 0.05) from 7.2 (Log CFU/g) on day 0 to 9.2 (Log CFU/g) on day 20, but the count of this lactic bacterium decreased significantly (*p* ≤ 0.05) in samples containing 1 to 3% REO (Figure 3c). The control had the highest number of *L. fermentum*, while the lowest count was detected in Sarshir containing 3% REO. From the fourth day onwards, the number of *L. fermentum* was significantly (*p* ≤ 0.05) different in samples containing different amounts of REO. According to Figure 3d, the number of *L. pentosus* in the control sample increased significantly (*p* ≤ 0.05) during cold storage and reached from 7.2 (Log CFU/g) on the day zero, to 9.3 (Log CFU/g) on the 20th day of the storage period. On the other hand, the numbers of this bacterium in the samples containing REO decreased significantly (*p* ≤ 0.05). The control and the sample containing 3% REO had the highest and lowest numbers of *L. pentosus*, respectively. The difference between the samples containing REO was also significant (*p* ≤ 0.05) in terms of the count of *L. pentosus* during the cold storage period. In general, increasing the concentration of REO caused a significant (*p* ≤ 0.05) decrease in the number of pathogenic and spoilage bacteria during the cold storage period. In addition, the highest reduction in the number of bacteria in the samples containing REO occurred in the interval from 16 to 20 days.

Most of the antimicrobial effect of EOs are related to phenolic components, which usually change the permeability and integrity of the bacterial cell membrane [27]. El-Sayed and Youssef [28] attributed the high antibacterial activity of dry rosemary to the high amounts of rosmarinic acid, caffeic acid and flavones.

The inhibition of two pathogens *S. aureus* and *S.* Typhi under the effect of REO has been found by Puvača et al. [29]. Kamel et al. [30] reported the antimicrobial effect of REO in two concentrations of 0.5 and 0.7% against *S. aureus* in stirred-like yoghurt. They attributed the potent antimicrobial activities of REO to the presence of monoterpenes such as alpha-pinene, 1,8-cineole, and borneol. Kontogianni et al. [31] used WPC active film containing rosemary ethanolic extract to coat soft cheese, and observed that the active film has an inhibitory effect against *S. aureus* compared to the control film. 

EOs have the potential to inhibit food-spoilage lactic acid bacteria (LAB). For instance, the inhibitory effects of some EOs on *Pediococcus acidilactici*, *Lactobacillus buchneri* and *Leuconostoc citrovorum* have been reported [32]. In the study of Moro et al. [33] they used REO in sheep milk cheese and did not observe any significant decrease in the number of LAB during cold storage. Using rosemary and oregano EO (OEO) in the slurry of fresh coalho cheese led to a reduction in *L. lactis* and *L. cremoris* during 72 h of cold storage [34]. Diniz-Silva et al. [35] reported a delay in increasing the numbers of probiotic *L. acidophilus* LA-5 in Minas cheese containing rosemary and OEO during 21 days of refrigeration. They attributed this delay to higher terpene EO concentrations in the cheese at the beginning of the ripening period. According to their report, eucalyptol, camphor, α-pinene, β-pinene, myrcene, ρ-cymene, α-o-cimene, isoborneol, α-terpinenol and caryophyllene constituted the highest amounts of terpenes in Minas cheese containing OEO and REO during the storage period.

### 2.5. Antioxidant Activity of REO in Sarshir

**Figure 4:**
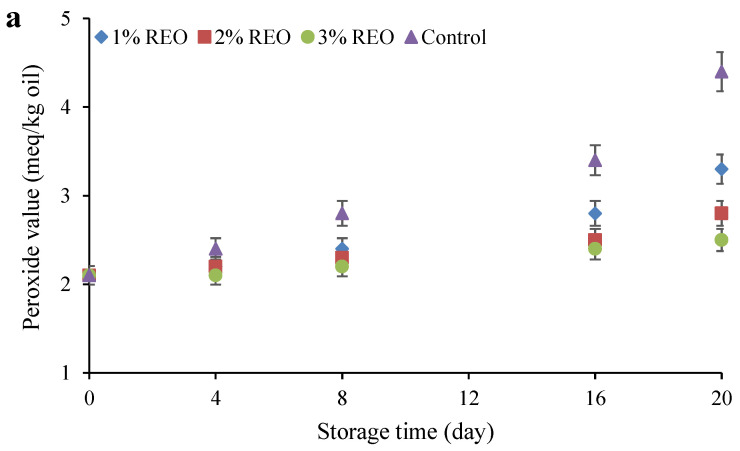

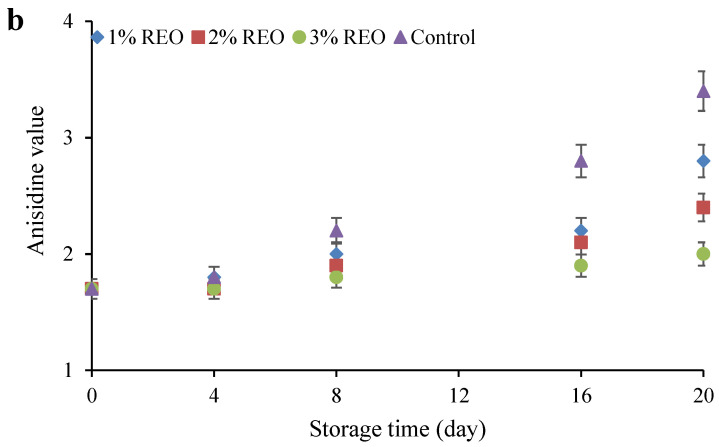
Changes in the peroxide (**a**) and anisidine (**b**) values of Sarshir during the cold storage period.

To evaluate the progress of lipid oxidation in food products, two indices of peroxide and anisidine are used. The peroxide index determines the primary oxidation of lipids. Figure 4a shows the changes in the peroxide value of the samples during refrigerated storage. As can be observed, the peroxide values of all samples increased with time, and this increase was significant (*p* ≤ 0.05) at the end of the storage period compared to the beginning. The highest degree of lipid oxidation was found in the control and Sarshir containing 3% REO had the lowest peroxide value. By increasing the concentration of REO from 1 to 3%, the peroxide value of the samples diminished, which was significant (*p* ≤ 0.05) at the end of the storage time.

The anisidine value determines the concentration of secondary oxidation products. Figure 4b shows the changes in anisidine values of the samples as a function of refrigeration time. Accordingly, increasing the storage time caused significant (*p* ≤ 0.05) increases in the anisidine values of the samples. The highest anisidine value was measured in the control, while the sample containing 3% REO had the lowest anisidine value. With increasing concentration of REO, the anisidine value of the samples containing essential oil decreased, which was especially significant (*p* ≤ 0.05) at the end of the cold storage period.

Oxidation of lipids negatively influences the quality of dairy products rich in fat by producing aldehydes, ketones and fatty acid hydroperoxides during refrigeration [36,37]. Free fatty acids resulting from triglyceride hydrolysis during cold storage accelerates the degradation of peroxides into oxidation products [38]. The rearrangement of double bonds during the formation of hydroperoxides from unsaturated fatty acids leads to the production of conjugated dienes. For this reason, the oxidation parameters of the peroxide value, anisidine value and conjugated diene value increase during the oxidation of lipids that occurs during storage [39,40]. Hashemi, Mousavi Khaneghah, Kontominas, Eş, Sant’Ana, Martinez and Drider [12] reported an increase in the peroxide and anisidine values of different Sarshir samples during a ten-day storage period. The increase in these indices in other dairy products, including cheese [41], yoghurt [42,43], ice cream [38], butter [44], Doogh [40] and yoghurt dessert [37], have also been reported during the storage period.

Milk naturally has a series of antioxidant compounds that are capable of scavenging superoxide, peroxide and hydroxyl radicals [41]. Phenolic compounds are believed to be responsible for the antioxidant activity of plant materials. Phenolic antioxidants act as absorbers by donating a proton, thus inhibiting the auto-oxidation process. Olmedo et al. [45] used REO in the production of flavoured cheese and found that cheese containing REO had better resistance to lipid oxidation than the control sample. They stated the high content of phenolic compounds in the EO as the reason for its high antioxidant activity. Branciari et al. [46] found that the rosemary phenolic components significantly enhanced the antioxidant capacity of pecorino cheese and effectively inhibited its lipid oxidation. Qiu et al. [47] found that the addition of rosemary extract significantly decreased the value of peroxide in cow’s milk compared to the control. They attributed the inhibition of lipid oxidation in fish oil-enriched milk products to the phenolic compounds present in rosemary extract. Ullah et al. [48] also reported that the addition of dry rosemary to cottage cheese had a high antioxidant effect due to the high amounts of rosmarinic and caffeic acids, flavones and phenolic diterpenes.

## 3. Materials and Methods

### 3.1. Bacterial Strains

Spoilage and pathogenic bacteria in Sarshir including *L. pentosus* PTCC 1872, *L. fermentum* PTCC 1638, *S. aureus* PTCC 1826 and *S.* Typhi PTCC 1609 were purchased from the Persian Type Culture Collection (IROST, Tehran, Iran). *L. pentosus* and *L. fermentum* were activated in MRS broth medium for 24 h at 37 °C and *S.* Typhi and *S. aureus* were activated in nutrient broth and tryptic soy broth medium, respectively, for 24 h at 37 °C.

### 3.2. REO Extraction and Chemical Characterization

Dried plant leaves were purchased from a medicinal plant shop in Tehran (Tehran, Iran). About 100 g of the plant was poured into a volumetric flask and then 400 mL of distilled water was added to it. After that, REO extraction was done for 110 min using a Clevenger apparatus. The obtained REO was dehydrated with sodium sulphate and then stored at 4 °C in dark glasses [49]. The yield of REO was 1.7% (*v*/*w*).

For GC/MS analysis (Agilent 7990, Santa Clara, CA, USA), an HP-5 MS capillary column (30 m × 0.25 mm i.d., 0.25 μm f.t.) was applied to identify the components. Helium was used as a carrier gas at a rate of 5 mL/min. Thermal programming of the column was performed from 60 to 240 °C with an increase rate of 4 °C/min. The temperature of the injection part was set at 240 °C. The amount of injection was 0.1 µL [50]. The REO was diluted with n-hexane (1/10, *v*/*v*) and a volume of 0.1 µL was injected with the split ratio: 1/10. The compounds were identified by comparing their mass spectral fragmentation patterns with those of similar compounds in the database (Wiley/NBS library) and with mass spectra literature data [51].

### 3.3. Antimicrobial and Antioxidant Activities of REO

To perform this test, 100 µL of the suspension of each bacterium (~6.1 log CFU/mL) was poured into the culture medium. Mueller–Hinton agar was used for *S.* Typhi and *S. aureus*, and Mueller–Hinton agar +10% MRS medium was used for *L. pentosus* and *L. fermentum*. About 10 µL of the REO was poured onto a sterile filter disc (diameter 6 mm) and placed in the culture medium. Then the plates were incubated for 18 to 24 h at a temperature of 37 °C and the diameter of the inhibition zone was measured [52].

DPPH was used to measure the antioxidant properties of the EO. For this purpose, 50 µL of different concentrations of EOs were mixed with 5 mL of methanolic DPPH solution (0.004%) and after 30 min of storage at room temperature and in a dark place, the absorbance of the samples was read at a wavelength of 517 nm [53].

### 3.4. Preparation of Sarshir Samples

Sarshir was purchased from a dairy shop in Shiraz city (Fars, Iran) and then heated at 90 °C for 5 min. After cooling, the target bacteria (*L. pentosus*, *L. fermentum*, *S.* Typhi and *S. aureus*) were inoculated separately to the Sarshir (~7.1–7.3 log CFU/g), and then the REO was added to the samples in concentrations of 1 to 3% (*v*/*w*). The samples were stored in closed glass containers for 20 days at 4 °C and microbial and oxidation tests were performed every 4 days.

### 3.5. Enumeration of Bacteria

To enumerate the bacteria, first the Sarshir samples were diluted using peptone water and then poured into a suitable culture medium. MRS agar medium was used for *L. pentosus* and *L. fermentum*, and nutrient agar and trypticase soy agar were used for *S.* Typhi and *S. aureus*, respectively. The plates were incubated at 37 °C for 24 to 48 h.

### 3.6. Peroxide Value (PV) and Anisidine Value (AnV) Determination

First, Sarshir lipid was extracted using a mixture of methanol/chloroform (1:1, *v*/*v*) according to the method of Bligh and Dyer [54]. Then, the ferric-thiocyanate method, proposed by [55], was used to measure the peroxide value. In order to measure the anisidine value, the absorbance of the solution containing lipid, isooctane and *p*-anisidine reagent was read at 350 nm [56].

### 3.7. Statistical Analysis

Statistical analyses were carried out using ANOVA and significant differences at *p* < 0.05 were measured using *t*-tests and Duncan’s multiple range tests using the SPSS program (v. 20.0 for Windows, SPSS Inc., Chicago, IL, USA).

## 4. Conclusions

Health problems caused by lipid oxidation have attracted the attention of researchers. Considering the high-fat content of Sarshir, it is very important to inhibit its lipid oxidation. Based on the results of the present study, REO was able to delay the lipid oxidation of Sarshir due to its antioxidant compounds including α-pinene, 1,8-cineole, camphor, camphene and limonene. Increasing the concentration of REO also led to a decrease in the peroxide and anisidine values in the samples. In addition, the numbers of pathogenic and spoilage bacteria in samples containing REO were reduced during the cold storage period, which indicates the antimicrobial effects of REO compounds. Considering the destructive effects of thermal processes on high-fat products and the intensification of oxidation reactions, it seems necessary to use alternative and economic processes. The use of available, non-toxic and inexpensive EOs, which contain high amounts of phenolic compounds, can improve the functional properties of the product while reducing the negative impact of traditional processes. It is suggested to use the combination of EOs and other green technologies such as fermentation in future research.

## Figures and Tables

**Table 1:** Chemical constituents of REO.

N.	Constituents	Retention Index (RI)	Relative Percentage (%)
1	Tricyclene	928	0.4
2	α-Thujene	933	0.05
3	α-Pinene	941	24.62
4	Camphene	955	8.1
5	Verbenene	971	1.22
6	Sabinene	980	0.12
7	β-Pinene	982	1.09
8	Octen 3-ol	983	0.1
9	3-Octanone	987	1.3
10	Myrcene	992	4.1
11	3-Octanol	994	0.1
12	6-Methyl-5-Hepten-2-ol	996	0.1
13	α-Phellandrene	1005	0.1
14	δ-2-Carene	1006	0.1
15	α-Terpinene	1021	1
16	*para*-Cymene	1031	1
17	Limonene	1034	6.1
18	1,8-Cineole	1039	14.1
19	Z-β-Ocimen	1044	0.06
20	Benzene acetaldehyde	1049	0.1
21	γ-Terpinene	1066	1.1
22	Cis-Sabinene hydrate	1081	0.06
23	Terpinolene	1095	1.1
24	Linalool	1107	2.13
25	Phenyl ethyl alcohol	1111	0.022
26	*endo*-Fenchol	1125	0.05
27	Chrysanthenone	1139	0.6
28	Camphor	1157	13.53
29	Camphene hydrate	1159	0.07
30	Trans-Pinocamphone	1171	0.45
31	Pinocarvone	1174	0.17
32	Borneol	1177	2.13
33	*n*-Nonanol	1178	0.56
34	Terpine-4-ol	1190	0.62
35	α-Terpineol	1194	1.09
36	Myrtenol	1207	0.3
37	Verbenone	1220	5.21
38	Citronellol	1233	0.1
39	Carvone	1248	0.1
40	Geraniol	1259	0.3
41	Bornyl acetate	1298	3.4
42	*para*-Cymene-7-ol	1299	0.1
43	Trans-Sabinyl acetate	1302	0.04
44	Tridecane	1305	0.09
45	Neryl acetate	1373	0.1
46	Geranyl acetate	1388	0.1
47	β-Caryophyllene	1428	1.8
48	Geranyl acetone	1462	0.1
49	α-Humulene	1464	0.27
50	Caryophyllene oxide	1590	0.11
51	Eicosane	2007	0.3
	Total identified		99.96

## Data Availability

Research data are not shared.
